# A Novel Method of Impeller Blade Monitoring Using Shaft Vibration Signal Processing

**DOI:** 10.3390/s22134932

**Published:** 2022-06-29

**Authors:** Jindrich Liska, Vojtech Vasicek, Jan Jakl

**Affiliations:** NTIS—New Technologies for the Information Society, Faculty of Applied Sciences, University of West Bohemia, Univerzitni 8, 301 00 Plzen, Czech Republic; jinliska@ntis.zcu.cz (J.L.); jjakl@ntis.zcu.cz (J.J.)

**Keywords:** steam turbine, impeller blade, vibration, monitoring, diagnostics, algorithm, signal processing

## Abstract

The monitoring of impeller blade vibrations is an important task in the diagnosis of turbomachinery, especially in terms of steam turbines. Early detection of potential faults is the key to avoid the risk of turbine unexpected outages and to minimize profit loss. One of the ways to achieve this is long-term monitoring. However, existing monitoring systems for impeller blade long-term monitoring are quite expensive and also require special sensors to be installed. It is even common that the impeller blades are not monitored at all. In recent years, the authors of this paper developed a new method of impeller blade monitoring that is based on relative shaft vibration signal measurement and analysis. In this case, sensors that are already standardly installed in the bearing pedestal are used. This is a significant change in the accessibility of blade monitoring for a steam turbine operator in terms of expenditures. This article describes the developed algorithm for the relative shaft vibration signal analysis that is designed to run in a long-term perspective as a part of a remote monitoring system to track the natural blade frequency and its amplitude automatically.

## 1. Introduction

The growing demand for higher power generation brings the need for increasing reliability and also the efficiency of steam and gas turbines together with ensuring safe operation. Early detection of potential fault is the key to avoid the risk of unexpected turbine outages and to minimize profit loss. One of the critical parts of the turbine is the blades of the last stages of the low-pressure turbine, where acting forces are essential in terms of the residual life. This is the key part that needs to be monitored in a long-term perspective to ensure the warning if any potential fault is present. In general, the existing approaches to blade vibration monitoring are based on contact or non-contact measurement.

The contact approach is based on strain gauging. This provides information about the mechanical stress on the blade surface. Because of the contact principle, it allows to obtain very accurate stress sampled at a high rate. This is useful in the signal analysis that follows. However, it is also necessary to transmit the electric signal outside the rotating part of the turbine [1]. In addition, the sensors must be resistant to the extreme conditions that occur in the flow section of the turbine. These are the reasons the strain gauge is unsuitable for the long-term monitoring of impeller blades.

The non-contact method can be represented by the popular blade tip-timing method (BTT) [2]. Sensors, in that case, are built right into the stator body to detect the times of the arrival of the blades. Detected times of arrival are further processed to evaluate the blade tip deflection. Vibrations of each blade are sampled once per revolution and, thus, proper diagnostics and condition monitoring require specialized signal processing techniques to be applied. In principle, this method is suitable for long-term monitoring. However, the costs of specialized sensors and the need for their installation right into the stator body are the main reasons that BTT systems are not so widespread. Additionally, the installation costs are not negligible. It is even a frequent practice not to monitor the impeller blades at all.

Another technique for measuring blade vibrations mentioned in the literature is the use of a pressure sensor installed in the stator body [3,4]. In principle, the rotating blades cause pressure fluctuations in the inner part of the stator, especially between the tip of the blade and the wall of the stator. The frequency of this fluctuation is equal to the blade passing frequency (BPF). The location of installed sensors can be compared to the BTT method. Unlike BTT, the measurement is, in principle, not limited by the sampling frequency given by the shaft speed; however, the need for the installation of the specialized sensors right into the stator body remains. The impeller, as mentioned in the previous paragraph, causes pressure fluctuations on the inside of the stator by its rotation. The pressure fluctuation causes a force to act on the stator wall and, thus, the absolute stator vibrations can be used for blade vibration monitoring. The advantage of this approach is, in contrast to the pressure measurement inside the stator, the relative simplicity of the sensor installation. However, this approach does not provide the direct measurement of blade vibrations. The location of the accelerometer is usually above the impeller, or the standard sensors installed on bearing pedestals are used. In that case, the monitored BPF amplitude may change by propagating the vibrations through the material.

Recently, the use of torsional rotor vibrations in terms of blade vibration monitoring was published [5]. Torsional rotor vibrations can be characterized as angular oscillations of the shaft. In principle, the reference markers around the shaft are measured in each revolution. The reference can be the zebra tape or, for example, the teeth of the gear. The measured signal has a character of a pulse signal similar to times of arrival in the case of BTT. It turns out that the frequency spectrum of such a signal also contains information about blade vibrations. The physical principle of how the blade oscillations propagate to the measured signal is described in [6]. It states that the total moment of inertia given by the contribution from all blades must be large enough to be reflected on the shaft. It is obvious that the contributions of the individual blades cancel each other out in the total moment of inertia. The exception for the case of tangential natural vibrations of the blades, which can cause torsional vibrations of the shaft, is 0 nodal diameter, when all blades oscillate with the same phase. Until recently, only 0 nodal diameters of blade vibrations were captured using the torsional vibration measurement.

## 2. Method of Impeller Blade Monitoring

**Figure 1:**
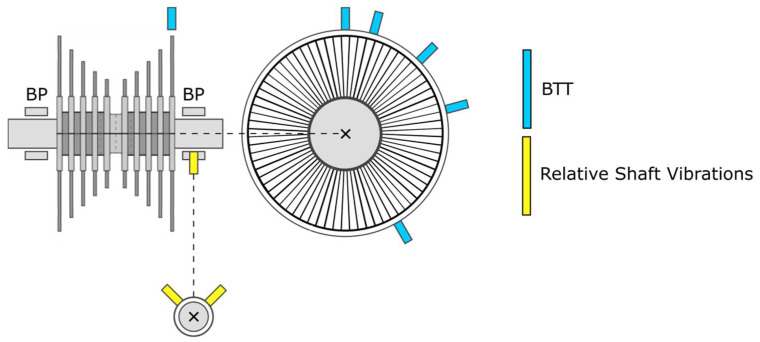
Low-pressure turbine scheme with the sensor location of BTT sensors and standard shaft vibration sensors.

A novel approach to impeller blade monitoring was developed in recent years by the authors of this paper. This approach is based on the evaluation of the blade vibrations using the shaft vibration signal analysis. The measurement of shaft vibrations is made using the standardly installed sensors placed in the bearing pedestals. This is a significant change in the accessibility of impeller blade monitoring for a steam turbine operator in terms of expenditures. There is no need to install any special sensor. Blade vibrations are evaluated using the already installed standard instrumentation and there is no need for the steam turbine outage. The sensor location for this approach together with the comparison with BTT sensors placing is illustrated using the low-pressure turbine scheme in Figure 1.

The blade vibration is present in shaft vibrations because of the bending moment acting on the shaft that is caused by blade axial oscillations. In fact, the blade vibrations are present in a form of two spectral components. The frequencies of these components are described by $f_{rot}\pm \;f_{n}$. This is due to an amplitude modulation with a suppressed carrier that occurs under the sensor during the rotation. These two components are the so-called lower sideband (LSB) and upper sideband (USB), as shown in Equations (1) and (2) respectively:

(1) $$\mathrm{USB}=f_{rot}+f_{n}$$

(2) $$\mathrm{LSB}=f_{rot}-f_{n}$$

**Figure 2:**
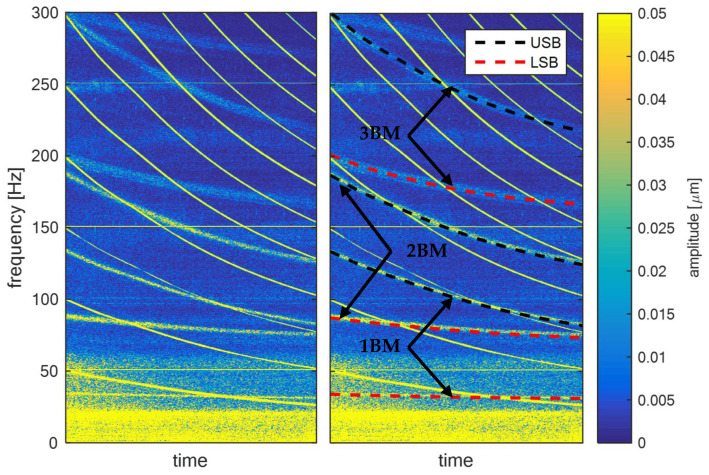
Spectrogram of shaft vibration signal from low-pressure turbine (**left**) with highlighted LSB and USB components (**right**).

The illustrations of blade components are illustrated in Figure 2, where on the left is the spectrogram of shaft vibration signal measured in real operation of the low-pressure turbine. On the right is the same spectrogram with highlighted LSB and USB components related to the blade vibration for the first three bending modes (BM). From this Figure, the presence of blade vibration components is clear.

The detailed description of this principle was published in the literature recently [7,8,9,10]. For proper impeller blade state diagnostics, the exact parameters of the blade components from the shaft vibration signal need to be obtained. Such a method is not published yet, so the purpose of this research was to develop and introduce the appropriate method for real-time blade monitoring using the shaft vibration signal from the long-term perspective. The description of this method is the main objective of this article.

**Figure 3:**
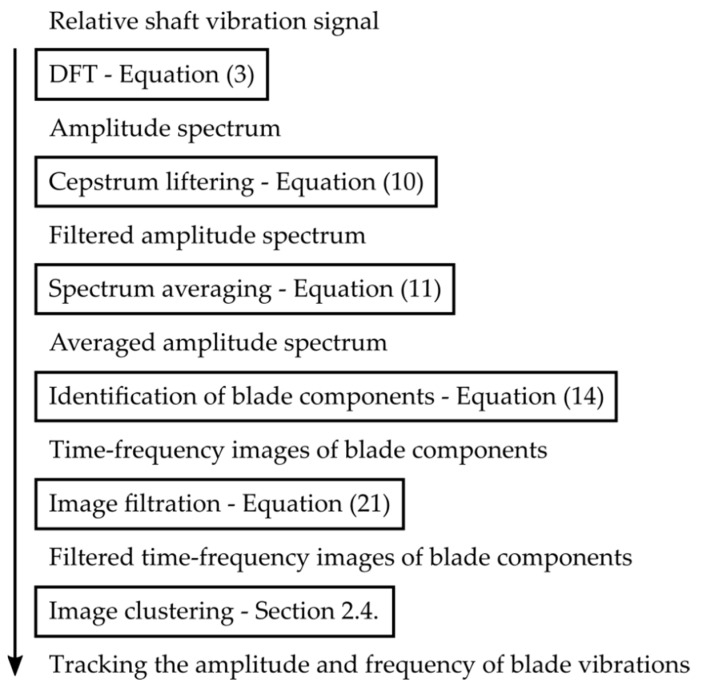
Flowchart of the developed method for online long-term blade vibration monitoring.

Firstly, the measured signal is pre-processed. This includes the signal filtering based on cepstral liftering and the spectrum time averaging in the spectral plane (flowchart in Figure 3) where the block schema of the proposed method is illustrated. The pre-processed signal is then analyzed and the blade components are identified, filtered in the time-frequency domain and clustered using the Euclidean distance. This algorithm is designed to run in a long-term perspective as a part of a remote monitoring system to track the natural blade frequency and corresponding amplitude automatically.

### 2.1. Cepstral Liftering of the Shaft Vibrations

The measured time signal of relative shaft vibrations is pre-processed first. The pre-processing is made in the frequency domain. For this purpose, the Discrete Fourier transform (DFT) is used because the measured signal is finite and discrete [11,12,13,14]. Formula (3) defines a spectrum of a signal x[n] sampled by frequency $f_{s}$, where n is the index of the signal sample:

(3) X[k]=DFT{x[n]}

At this point, it is also desirable to define a one-sided amplitude spectrum that will be used afterwards (Formula (4)). The spectral frequency corresponding to integer index k follows Formula (5), where L is the length of the signal.

(4) $$A\left[k\right]=\begin{array}{c} \;\;\left|X\left[k\right]\right|,\;\;\;\;k=0\\ 2\left|X\left[k\right]\right|,\;\;\;\;k>0 \end{array}$$

(5) $$f_{k}=k\cdot f_{s}/L$$

Measurement of the relative shaft vibration signal contains the noise that is unevenly distributed across the amplitude spectrum. To be able to identify the blade vibrations components using the following analysis, the signal noise needs to be filtered first. For this purpose, the cepstral analysis and spectral envelope calculation are used [15].

The cepstrum calculation is the basic method of cepstral analysis. The so-called real cepstrum that is defined by Formula (6) is an inverse Fourier transform (IDFT) of a logarithm of an amplitude spectrum of the sampled signal Formula (6).

(6) c[n]=IDFT{ln(|X[k]|)}

The cepstrum itself characterizes the periodicity of the amplitude spectrum. Low quefrencies represent the low-frequency periodicity in the spectrum and vice versa. This can be used for cepstrum liftering. It is a process of weighting the cepstrum by the lifter function. This is represented by Formula (7):

(7) $$c_{w}\left[n\right]=c\left[n\right]\cdot w\left[n\right]$$

Using liftering the unwanted cepstrum coefficients are suppressed. For the spectral envelope calculation, the lifter in a form of the Gauss function is used (Formula (8)). In that case, the high quefrencies are suppressed leaving just low quefrencies in cepstrum that can be used for the spectral envelope calculation (Formula (9)):

(8) $$w\left[n\right]=e^{-\frac{n^{2}}{2\sigma^{2}}}$$

(9) $$E\left[k\right]=e^{\mathrm{DFT}\left\{c_{w}\left[n\right]\right\}}$$

The level of the noise that is present in the measurement is then filtered simply by subtraction of the spectral envelope from the original amplitude spectrum (Formula (10)):

(10) $$X_{F}\left[k\right]=A\left[k\right]-2E\left[k\right]$$

### 2.2. Time Averaging of the Amplitude Spectrum

Filtering of the amplitude spectrum of the shaft vibration signal using the cepstrum calculation suppresses the mean value of the measurement noise on each spectral component. In addition to the mean value, the noise is also characterized by the variance in time that also needs to be suppressed. For this purpose, spectrum time averaging is used and so the frequency domain is extended into the time-frequency domain. The measured signal is divided into equidistant time series that are then transformed into a spectrum by DFT. The average amplitude spectrum is then defined using Formula (11) whereas N represents the number of averaged DFT spectrums. The time index l is tied up with the sampling frequency and parameter of the signal window shift in samples $\Delta_{w}$ from one DFT calculation to another (Formula (12)). For further use, the average amplitude spectrum is also indexed in time by index m similar to l. $t_{m}$ represents the time of average amplitude spectrum that is based on the shift in samples $\Delta_{m}$ in a similar manner to index l (Formula (13)):

(11) $$X_{F}^{T}\left[m,k\right]=\frac{1}{N}\left|\overset{m+N-1}{\underset{l=m}{\sum }}X_{F}\left[l,k\right]\right|$$

(12) $$t_{l}=\frac{l\cdot \Delta_{w}}{f_{s}}$$

(13) $$t_{m}=\frac{m\cdot \Delta_{m}}{f_{s}}$$

### 2.3. Identification and Filtration of the Blade Components

The pre-processed relative shaft vibration signal as a form of averaged cepstrum liftered amplitude spectrum is the input to the process of automatic identification of blade vibrations. The robustness of the calculation is taken into account because this algorithm is supposed to run on a long-term base with no user intervention. In this chapter, the process of the blade component identification together with its filtration is described. The expert task is also the proper selection of frequency interval to be monitored, defined by $f_{MIN}$ and $f_{MAX}$. The assumption for obtaining correct results for the identification process is that the monitored interval must not include the other excited components that are not related to the blade vibrations on such higher harmonics, etc.

The spectral components that are defined as blade vibration components in a shaft vibration signal spectra follow Formula (14). It is a set of $X_{F}^{T}\left[t_{m},f_{k}\right]$ that meets the conditions, its frequency is in the range defined by $f_{MIN}$ and $f_{MAX}$ (see $C_{1}$ (15)) and its amplitude is higher than the multiple of parameter $A_{TH}$, defined by the expert and the median of $X_{F}^{T}\left[t_{m},f_{k}\right]$ values around the analysed component, (see $C_{2}$ (16) and (17)). The size of the frequency interval for the median calculation represents parameter δ [Hz]:

(14) $$X_{NF}\left[t,f\right]=\left\{X_{F}^{T}\left[t_{m},f_{k}\right]|C_{1}\wedge C_{2}\right\}$$

(15) $$C_{1}:\;f_{k}\in \langle f_{MIN},f_{MAX}\rangle$$

(16) $$C_{2}:\;X_{F}^{T}\left[t_{m},f_{k}\right]>T\left[t_{m},f_{k}\right]$$

(17) $$T\left[t_{m},f_{k}\right]=A_{TH}\cdot \mathrm{med}\left(X_{F}^{T}\left[t_{m},f\right]|f\in \;\langle f_{k}-0.5\cdot \delta ,f_{k}+0.5\cdot \delta \rangle \right)$$

The components identified according to (14) are then filtered to suppress the events in a short-term horizon that do not follow the blade behavior. For this purpose, a subset of (14) for every component from (14) is defined according to Formula (18). This subset contains all $X_{NF}\left[t,f\right]$ from the rectangle R[t,f] defined in the time-frequency domain according to conditions $C_{3}$ and $C_{4}$ (see Formulas (19) and (20), respectively):

(18) $$R\left[t,f\right]=\left\{X_{NF}\left[t,f\right]|C_{3}\wedge C_{4}\right\}$$

(19) $$C_{3}:\;t\in \;\langle t,t+t_{FILT}\rangle$$

(20) $$C_{4}:\;f\in \;\langle f-0.5\cdot f_{FILT},f+0.5\cdot f_{FILT}\rangle$$

**Figure 4:**
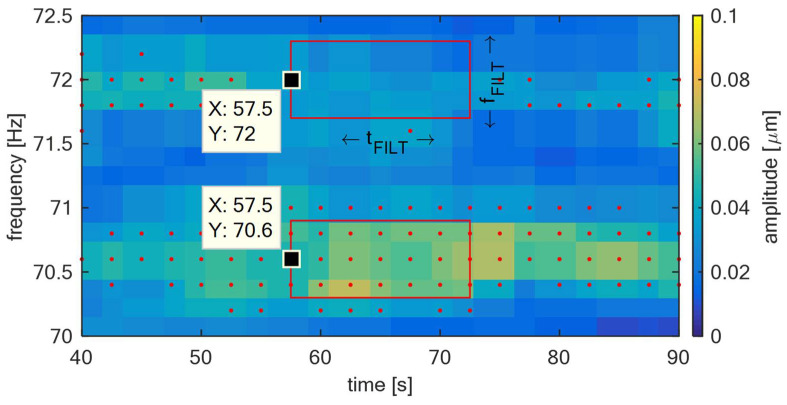
TG 250 MW—Filtering of identified components in the spectrum.

The example of this rectangle is illustrated for $X_{NF}\;\left[57.5,70.6\right]$ and $X_{NF}\;\left[57.5,72\right]$ in red in Figure 4. The meaning of the parameters $t_{FILT}$ and $f_{FILT}$ from this figure is evident—it is the time length and frequency length, respectively, of window rectangle R[t,f]. The red dots represent other identified $X_{NF}\left[t,f\right]$ according to the algorithm described in a previous part of this article. The filtration itself is defined according to Formula (21). The non-filtered $X_{NF}\left[t,f\right]$ is filtered if the number of $X_{NF}\left[t,f\right]$ in R[t,f] does not exceed the threshold $N_{TH}$. From Figure 4, it is obvious that $X_{NF}\left[57.5,70.6\right]$ becomes $X_{I}\left[57.5,70.6\right]$ and $X_{NF}\left[57.5,72\right]$ not because |R[t,f]|=0. This process filters randomly excited noise components that can randomly occur during the measurement in the short-term, making this algorithm more robust and to ensure that false alarms are not generated.

(21) $$X_{I}\left[t,f\right]=\left\{X_{NF}\left[t,f\right]|N_{TH}>\left|R\left[t,f\right]\right|\right\}$$

### 2.4. Clustering of the Blade Components

For practical use, the identified blade vibration components from the previous chapter need to be interpreted in an intelligible way. It is appropriate to represent each of the blade frequencies with one frequency and amplitude value that can be tracked over time, instead of many identified components. For this purpose, the identified blade components are clustered using cluster analysis [16].

This analysis groups identified components into sets according to a defined degree of similarity. The red components identified in the previous section and illustrated in Figure 4 are being called images of blade vibrations in the time-frequency plane of the relative shaft vibration signal. Using this terminology, the i-th image is indexed and abbreviated according to Formula (22). The degree of similarity of two images is based on their frequency distance. This can be defined by the Euclidean distance represented by operator d that is in form of the Formula (23) for one-dimensional space. This relation defines the distance between the i-th and j-th image $X_{I}$:

(22) $$X_{I}^{i}=X_{I}\left[t_{i},f_{i}\right]$$

(23) $$d\left(X_{I}^{i},X_{I}^{j}\right)=\left|f_{i}-f_{j}\right|$$

The clustering process used in this algorithm is defined according to the following sequence. In the first step of clustering at time $t_{1}$, image 1 is declared to be the first cluster (Formula (24)). The cluster is given by S and indexed by k. Each cluster can be represented by its center. The center of cluster $S_{k}$ is defined using Formula (25), where $N_{k}$ denotes the number of images $X_{I}$ assigned to the cluster $S_{k}$:

(24) $$X_{I}^{1}\in S_{1}$$

(25) $$\mu_{k}=\frac{1}{N_{k}}\underset{f\in S_{k}}{\sum }f$$

In the following j-th iteration of this algorithm, steps represented by Formulas (26)–(30) are being repeated for the analyzed $X_{I}^{j}$ image. The first procedure is to exclude the images from the cluster sets that are older than the time of the actual image for more than the forgetting factor $t_{TH}\;\left[s\right]$ (see Equation (26)). This step is important to allow the algorithm to capture any changes in blade natural frequency that occurs over the monitored period:

(26) $$S_{k}=S_{k}\setminus \left\{X_{I}|\;t<\left(t_{j}-t_{TH}\right)\right\}$$

Updating the cluster images is followed by the recalculation of their centers. The distance between the cluster centers and the j-th image $X_{I}^{j}$ is then determined. The number of the clusters is K. The nearest cluster to the j-th image is $I_{MIN}$ in a distance $M_{MIN}$ (see Formulas (27) and (28)). If the distance $M_{MIN}$ is less than the frequency threshold $f_{TH}$, then the j-th image is assigned to the $I_{MIN}$-th cluster (Formula (29)). Otherwise, a new cluster is established (Formula (30)) and the next iteration of the clustering process is executed. The choice of $f_{TH}$ allows to set the precision of spectral components that can be distinguished by the proposed algorithm:

(27) $$I_{MIN}=\underset{k}{\mathrm{argmin}}\;d\left(X_{I}^{j},\mu_{k}\right),\;k=1,\ldots ,K$$

(28) $$M_{MIN}=\underset{k}{\min}\;d\left(X_{I}^{j},\mu_{k}\right),\;k=1,\ldots ,K$$

(29) $$X_{I}^{j}\in S_{I_{MIN}},\;M_{MIN}\leq f_{TH}$$

(30) $$X_{I}^{j}\in S_{k+1},\;M_{MIN}>f_{TH}$$

## 3. Results

To demonstrate the described algorithm, the measurement made in the operation of TG 250 MW was used. The shaft vibration signal was captured by an eddy current displacement sensor that met the standard for use in practice. The measuring range of the sensor was from 0–2 mm and the sensitivity 8 V/mm. The data acquisition was made using the National Instruments hardware. The Chassis cDAQ-9189 equipped with an NI-9229 4 channel module was used. This module allowed to measure analog inputs with 24-bit resolution, the maximal sampling frequency of 50 kHz and an input voltage range from −60 V to 60 V. The shaft vibration signal was captured using the sensor installed in the front bearing pedestal of the low-pressure turbine. The last stage blades were shrouded on the tips and in the middle with a tie-boss. The distance between the bladed wheel and the sensor was 2.4 m.

**Figure 5:**
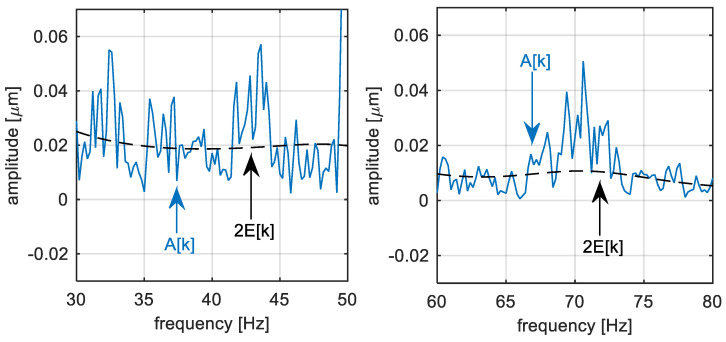
TG 250 MW—Discrete Fourier transform amplitude spectrum of the shaft vibration signal and its spectral envelope—liftered spectrum.

The example of DFT of the shaft vibration signal that was measured in the operation of TG 250 MW is illustrated in the form of an amplitude spectrum according to Equation (4) in Figure 5 in blue. The length of the analyzed signal was 5 s, the sampling frequency was 10240 Hz, the rotational frequency was 3000 rpm (nominal speed) and the Hanning window was used. In the signal spectrum, 1X frequency—50 Hz—and its integer multiplies were dominant. As well as those components, the blade vibration components can also be, principally, present in the signal. In Figure 5, there are two parts of the amplitude spectrum of the shaft vibration signal. At this point of the signal processing procedure, it is not clear if any of those two intervals also contain the blade vibrations or not. The calculated spectral envelope is illustrated by the dashed black line in Figure 5. For its evaluation, the parameter σ was set to 0.16 (see Equation (6)).

**Figure 6:**
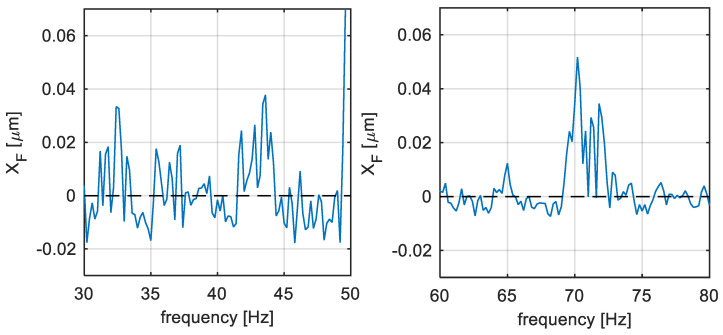
TG 250 MW—Filtered Discrete Fourier transform amplitude spectrum of the shaft vibration signal.

The step of the noise reduction according to Equation (10) is illustrated using Figure 6. It can be compared with the non-filtered amplitude spectrum in Figure 5. The noise level is suppressed by the spectral envelope. After this operation, the mean value of the spectral measurement noise on every spectral component is supposed to be suppressed.

**Figure 7:**
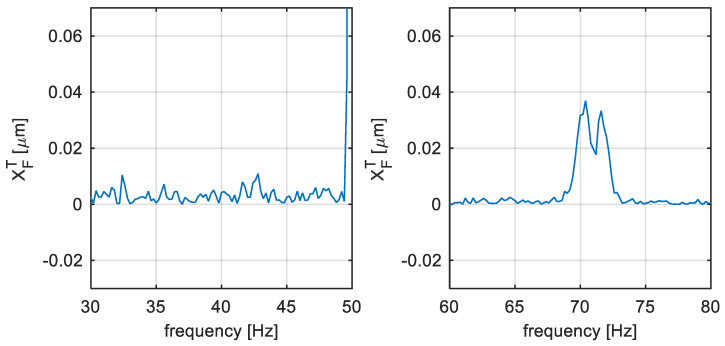
TG 250 MW—Time-averaged filtered amplitude spectrum of the relative shaft vibration signal.

Time averaging described in Section 2.2 was applied to the measurement, which is illustrated in Figure 7 in the frequency domain. The DFT is then defined in time instants corresponding to $\Delta_{w}=5f_{s}$ (Equation (12)). The number of spectrums for averaging satisfies the condition N=20, which means that the averaged spectrum characterizes 100 s of the relative shaft vibration signal.

In Figure 7, there are the same two amplitude spectrum intervals as in Figure 5 and Figure 6. It can be seen from this Figure that the range from 58–83 Hz contains the excited components, while the range from 25–50 Hz contains only the noise. This was not obvious in the previous steps illustrated by Figure 5 and Figure 6, respectively. The decision of whether the excitation is related to blade vibrations can be made by using a priori knowledge of the natural blade vibration frequencies standardly illustrated by the Campbell diagram [17]. Using this diagram, the approximate location of the blade natural frequency can be obtained and compared with the spectral components excited in a real measurement by the expert.

**Figure 8:**
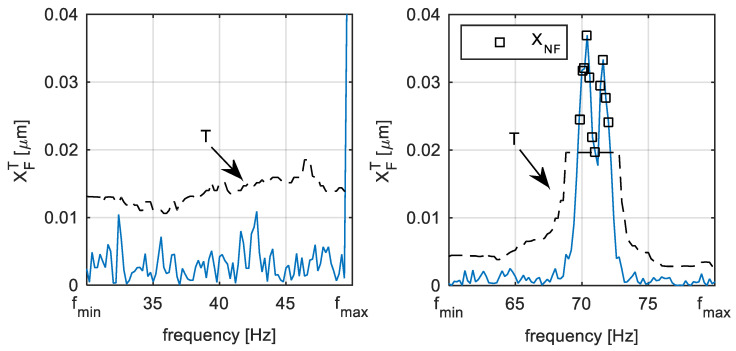
TG 250 MW—Blade component identification.

The example of the identification process is illustrated in Figure 8. There is the pre-processed signal of the shaft vibration in the form of an average amplitude spectrum, as it is illustrated in Figure 7. The dashed line represents the threshold defined in (17), that is in this case defined for $A_{TH}=5$ and δ=20 Hz. The black squares illustrate the identified components that exceed the threshold satisfying condition (16). It can be seen in Figure 8, that, if only the noise is present in the signal spectra monitoring range, there are no identified blade components and vice versa. The parameters should be set uniquely for each monitored frequency interval.

**Figure 9:**
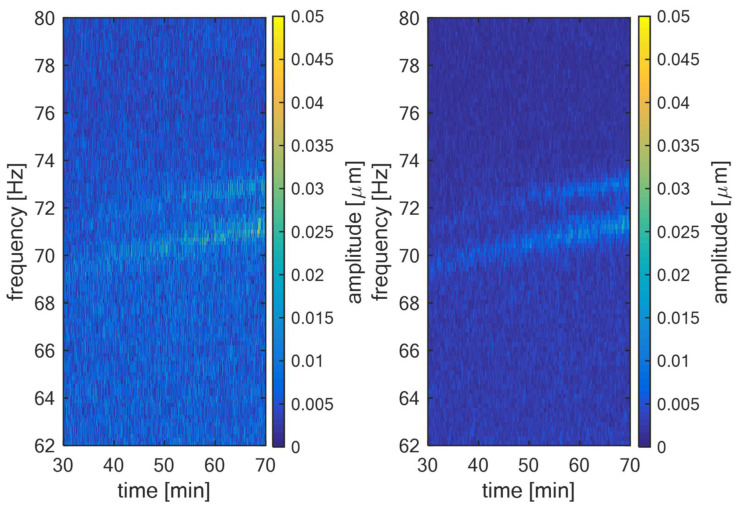
TG 250 MW—Amplitude spectrogram of the relative shaft vibration signal (**left**); averaged and filtered amplitude spectrogram of the same signal (**right**).

**Figure 10:**
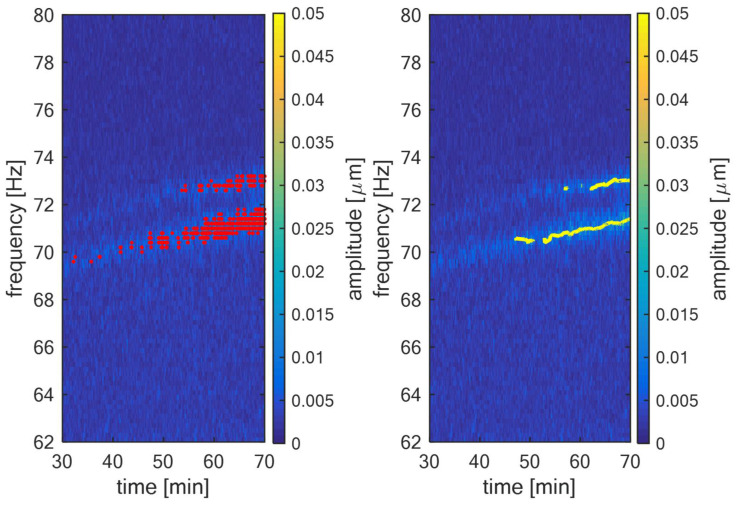
TG 250 MW—Identified blade vibration components in amplitude spectrogram of the relative shaft vibration signal (**left**); tracking of blade vibration components using the cluster analysis (**right**).

The example of the novel method for blade vibration monitoring that was described in this article is illustrated in Figure 9 and Figure 10. In Figure 9, left, there is the spectrogram of shaft vibration signal measured in the operation of TG 250 MW. The sampling frequency of the measurement was 10.24 kHz, the length of the Hanning window used in DFT was 5 s and $\Delta_{w}$ was $2.5f_{s}$. It can be seen that the relative shaft vibration signal itself is noisy. In Figure 9, right, there is a spectrogram of the same signal section, but this time pre-processed according to Equation (11). It can be seen that the noise was filtered and the blade components that were approximately 71 Hz and 72.5 Hz are more obvious, even with the naked eye. The parameters for pre-processing were: σ=0.16, $\Delta_{w}=\Delta_{m}$ and N=5, which corresponds to 15 s of signal for averaging. In fact, there are two blade components that increase their amplitude in time and also change their frequency because of the change in the steam turbine operating conditions.

In Figure 10, left, the identified blade components according to Equation (14) are illustrated in red. The parameters for this step were: δ=10 Hz, $A_{\mathrm{TH}}=3$, $f_{\mathrm{MIN}}=62$ Hz and $f_{\mathrm{MAX}}=80$ Hz. In Figure 10, right, there is the trend of the blade components after the cluster analysis according to Equation (25), illustrated in yellow. The parameters for this step were: $t_{FILT}=10$ s, $f_{FILT}=0.8$ Hz and $N_{TH}=8$. It can be seen that the developed algorithm effectively tracks both blade components that are present in the relative shaft vibration signal.

## 4. Discussion and Conclusions

The main objective of this work was to develop and validate the novel method for automatic long-term blade vibration monitoring using the relative shaft vibration signal analysis. This measurement uses the sensors that are standardly installed in the steam turbine. This is the main advantage over the other methods and principles of blade vibration monitoring. This approach is potentially interesting for steam turbine operation because there is no need of any special sensor installation. This article describes the developed method in detail. The measured vibration signal of shaft vibrations is firstly pre-processed. The spectral noise is filtered using the cepstrum analysis described in Section 2.1. Then, the spectral components are averaged in time to reduce the noise variance (see Section 2.2). After that, the pre-processed signal is used to identify the blade components that are excited according to the proposed identification rule described in detail in Section 2.3. The cluster analysis is the last step, which is used to merge blade components with close identified frequencies (see Section 2.4). Those frequencies may be tracked over time together with corresponding amplitudes and may be compared with the nominal values. If the differences reach the maximal allowed limit, then the alarm is triggered, meaning the blade state is in a non-standard state.

Another benefit of this approach is the easy acquisition of the natural frequencies of the installed blades from real operation without the need for outage. By simply connecting to existing standard instrumentation, the application can evaluate the actual natural frequencies of the blade, which differ slightly from numerical calculations. The turbine operator can include this information to minimize the operation time for the rotational frequency, or its integer multiple is close to any of the resonant frequencies of the blades that was evaluated using proposed approach.

The algorithm itself was tested and validated on the measurement data from the turbo generator of 250 MW. The validation of this algorithm is illustrated in the figures and spectrograms in this article. After the validation phase, the algorithm was implemented into the online monitoring and diagnostics system available for commercial purposes, which is currently monitoring the operation of the 215 MW turbo generator. Further research in this field will be focused on the specification of other diagnostic indicators that will extend the possibilities of the comparison between the actual turbo-generator operation and nominal state based on the identified blade components in an even more sophisticated way. Further research could be focused on how to evaluate the phase of both spectral components of the blades in addition to amplitude and frequency. This could even help to localize where the blade excitation is applied around the blade wheel.

## Data Availability

Not applicable.
